# Social Isolation in the Elderly: The Neglected Issue

**Published:** 2019-02

**Authors:** Ali SEYFZADEH, Mansoor HAGHIGHATIAN, Aliasghar MOHAJERANI

**Affiliations:** Department of Sociology, Dehaghan Branch, Islamic Azad University, Dehaghan, Iran

## Dear Editor-in-Chief

Social isolation is defined as discontinuing actual relations with society members, groups and communities, which leads to weakened or discontinued joining and participating in official and non-official groups. In fact, this term is interpreted as lack of or weak social connections and friendships, as well as correlation with official and non-official groups (1). In the literature of social sciences, isolation is recognized as the actual condition of withdrawal and exclusion from interactions and social spaces. In the modern world, creation and maintenance of social of relationships have been more complicated, increasing the risk of social isolation among various populations. Experience of loneliness and isolation is not equal during life. Generally, the elderly feel lonelier and are more isolated due to fatal events, such as retirement or losing loved ones (2).

Evaluation of social isolation among the elderly is crucial due to several reasons. Firstly, prevalence of social isolation increases by aging. Older adults often have smaller social networks and are more prone to loneliness. This might be partially due to the experience of deprivation and higher possibility of living alone among this population (3). Given the growing population of elderly and experience of aging by our country, it is significantly important to evaluate the social needs and communication network of the elderly. In Iran, 7.5% of the total population were elderly aged >65 yr in 2011 according to general census, which shows an increase of 0.5% compared to the census in 2006. Mean social isolation of the elderly was about 62% in Tehran, demonstrating the extensive expansion of social isolation of this age group in Tehran (4). In fact, social isolation threats the elderly more than diseases and even poverty. This feeling of loneliness might be an important factor in emergence of increase of other diseases in these individuals. One of the significant and effective factors for physical and mental health of the elderly is social participation, which leads to attention to the rights of the elderly, maintaining social order and improving the quality of life of these individuals. Attendance of the elderly in meaningful activities (generally in the form of friendship and organization participation) is one of the key elements of improving the quality of life and general health of these individuals, which leads to reduced disabilities during aging (5). In addition, social participation prevents psychological problems during this period of life since most of the elderly are faced with social isolation when separated from their families and society. Moreover, one of the fundamental factors for dealing with social isolation is educational intervention, which can alleviate social isolation and its unfavorable impacts on the elderly.

Notifications are announced on the risk of increased social isolation of the elderly and methods to reduce these factors in this vulnerable social class in order to have more active elderly with a higher level of health. Generally, recognition of predictive variables of social isolation in the elderly is a step toward successful decrease of the feeling of loneliness and its serious outcomes in this vulnerable social class.
